# Comparison of apoA-I helical structure and stability in discoidal and spherical HDL particles by HX and mass spectrometry

**DOI:** 10.1194/jlr.M034785

**Published:** 2013-06

**Authors:** Palaniappan Sevugan Chetty, David Nguyen, Margaret Nickel, Sissel Lund-Katz, Leland Mayne, S. Walter Englander, Michael C. Phillips

**Affiliations:** * Lipid Research Group, Gastroenterology, Hepatology, and Nutrition Division, Children's Hospital of Philadelphia, Philadelphia, PA 19104-4318; and; † The Johnson Research Foundation, Department of Biochemistry and Biophysics, Perelman School of Medicine at the University of Pennsylvania, Philadelphia, PA 19104-4318

**Keywords:** amphipathic α-helix, cholesterol, lipoprotein, phospholipid, hydrogen exchange

## Abstract

Elucidation of apoA-I secondary structure in spherical plasma HDL particles is essential for understanding HDL structure and function at the molecular level. To provide this information, we have applied hydrogen exchange (HX) and mass spectrometry methods to compare apoA-I secondary structure in discoidal (two apoA-I molecules/particle) and spherical (five apoA-I molecules/particle) HDL particles. The HX kinetics indicate that the locations of helical segments within the apoA-I molecules are the same in both discoidal and spherical HDL particles (approximately 10 nm hydrodynamic diameter). Helix stabilities in both types of particles are 3–5 kcal/mol, consistent with the apoA-I molecules being in a highly dynamic state with helical segments unfolding and refolding in seconds. For the spherical HDL, apoA-I fragments corresponding to residues 115–158 exhibit bimodal HX kinetics consistent with this segment adopting an inter-converting (on the timescale of tens of minutes) helix-loop configuration. The segment adopting this configuration in the 10 nm disc is shorter because the surface area available to each apoA-I molecule is apparently larger. Loop formation in the central region of the apoA-I molecule contributes to the ability of the protein to adapt to changes in available space on the HDL particle surface. Overall, apoA-I secondary structure is largely unaffected by a change in HDL particle shape from disc to sphere.

Apolipoprotein (apo)A-I is the principal structural component of high density lipoproteins (HDLs) and, as such, determines the structure of most species of HDL particles. Understanding how apoA-I performs this function requires knowledge of its organization on HDL particles of different sizes and shapes. The structures of reconstituted discoidal HDL particles which are similar to nascent HDL are the best defined. In particular, such particles with diameters of ∼10 nm have been shown to comprise a segment of phospholipid (PL) bilayer stabilized by two apoA-I molecules arranged in an anti-parallel double-belt conformation around the edge of the disc (1, 2). The intermolecular apoA-I contacts have been characterized using chemical cross-linking and mass spectrometry (MS) [for reviews, see (3–6)]. Hydrogen exchange (HX)-MS measurements have located the segments of α-helix within the apoA-I molecules and determined the stabilities of these helices (7). Most HDL particles in plasma are spherical because they contain a core of cholesteryl ester molecules created by the activity of lecithin-cholesterol-acyltransferase (LCAT). Chemical cross-linking studies have shown that the intermolecular contacts characteristic of the double-belt arrangement are maintained in spherical HDL particles of different sizes (8, 9). The transition from discoidal to spherical shape is accommodated by bending of the apoA-I molecules to form a trefoil structure (8). A related, less symmetric, structure has been proposed based on small angle neutron scattering studies of spherical HDL particles (10). The above studies suggest that the overall protein-protein contacts of the apoA-I molecules on discoidal and spherical HDL particles are the same. However, it is not known if apoA-I secondary structure is the same on the two types of particles. Earlier work has given conflicting evidence related to this point. Thus, the α-helix content and tryptophan fluorescence properties are similar for discs and spheres of the same size (11). However, on the other hand, the microenvironments of apoA-I lysine residues (12), protein charge (13), sensitivity to proteases (14), and apoA-I intermolecular separations (15) are different on the two types of particles.

To better understand the structure of apoA-I and to resolve the above issues, we have compared in detail the secondary structure of apoA-I on spherical and discoidal HDL particles. This task involved extending our previous HX-MS studies of apoA-I helix structure and stability in the lipid-free state (16, 17) and in discs of different sizes (7) to plasma HDL containing only apoA-I (LpA-I). These spherical particles which contain apoA-I as essentially their only protein constituent (9) comprise ∼25% of plasma HDL. The results show that the apoA-I intramolecular helix locations and stabilities are largely retained upon HDL transformation from a disc to a sphere of the same size. The exception is that the number of amino acids that form a loop segment located near the center of the molecule is greater in the spherical LpA-I particle, apparently the result of an increase in apoA-I surface packing density.

## EXPERIMENTAL

### Materials

MS grade formic acid and acetonitrile were obtained from Mallinckrodt Baker. D_2_O and porcine pepsin were purchased from Sigma. The pepsin was immobilized on Poros AL column packing according to the supplier's instructions (Applied Biosystems) and packed into an HPLC guard column (2 × 20 mm). 1-Palmitoyl-2-oleoyl-*sn*-glycero-3-phosphocholine (POPC) (>99% purity) was obtained from Avanti Polar Lipids while sodium cholate was bought from Sigma. Nondenaturing gradient polyacrylamide gels (4–20%) for estimating lipoprotein and protein sizes were provided by Invitrogen. Human apoA-I was purified as described previously (13).

### Methods

#### HDL preparation.

Homogeneous reconstituted 10 nm discoidal HDL was prepared from POPC and apoA-I by cholate dialysis (7, 18). Human plasma HDL (1.066–1.21 g/ml) was isolated by sequential ultracentrifugation and spun a second time to minimize albumin contamination. LpA-I was isolated from this HDL by covalent chromatography on thiopropyl Sepharose (9, 19). The LpA-I samples obtained in this fashion contained <5% of apoA-II as assessed with SDS-PAGE gels. The HDL preparations were purified to homogeneity by size-exclusion chromatography on two Hi Load Superdex 200 (GE Amersham Biosciences) columns (60 × 1.6 cm) linked in series. The columns were calibrated so that the hydrodynamic diameters of the HDL particles could be determined (20). The spherical HDL particles were also characterized with respect to size by nondenaturing 4–20% polyacrylamide gradient gel electrophoresis (21). The protein contents of the HDL preparations were determined using a modified Lowry assay (22). The PL content was determined by extracting the HDL lipids with the Bligh-Dyer procedure (23) and assaying for inorganic phosphorus (24). An HPLC analysis was used to separate and determine the amounts of different classes of PL (25). A gas-liquid chromatographic method was employed to measure the cholesterol and cholesteryl ester (26), and triglyceride was determined with an enzymatic assay kit (Thermo Fisher Scientific Inc.).

#### Circular dichroism.

Far-ultraviolet circular dichroism (CD) spectra of lipid-free apoA-I discoidal and spherical HDL were acquired using a Jasco J810 spectropolarimeter as described (27).

#### apoA-I cross-linking.

The number of apoA-I molecules in discoidal and spherical HDL particles was estimated by chemical cross-linking using bis(succinimidyl propionate) (BS^3^) which has a 12 Å spacer length. The cross-linking was performed as described earlier at a 1:50 protein:cross-linker (mol:mol) ratio (9). After cross-linking, the non-cross-linked HDL and cross-linked discoidal and spherical HDL samples were analyzed in 4–20% SDS-PAGE gels to estimate the size distribution of the cross-linked apoA-I molecules. MALDI-TOF mass spectrometry of cross-linked apoA-I in 10 nm spherical LpA-I HDL particles and discoidal HDL particles was performed with a Bruker Daltonics Microflex MALDI-TOF mass spectrometer using sinapinic acid as the MALDI matrix (10). The HDL samples were cross-linked by incubation at a 1:10 apoA-I:BS^3^ (mol:mol) ratio for 5 min at room temperature and quenched by addition of NH_4_HCO_3_ to a final concentration of 100 mM. The spectra were acquired in the linear mode and calibrated by two-point external calibration with human apoA-I (average mass = 28,079 Da) and IgG1 (AB SCIEX, Framingham, MA) (average mass = 148,500 Da). The acquired mass spectra were smoothed with the analysis software (Flex Analysis) provided by the instrument manufacturer.

#### Hydrogen exchange and mass spectrometry.

Hydrogen-deuterium exchange coupled with fragment-separation (28) and mass spectrometry analysis (29–31) was used to study the secondary structure of apoA-I in 10 nm discoidal and spherical HDL particles. The fragment-separation system consisted of a box maintained at 0°C with an immobilized pepsin column, a C18 trap, and an analytical C18 column connected in the order listed; for more details on the fragment separation system see (31). The outlet from the C18 analytical column was connected online to a LTQ Orbitrap XL mass spectrometer (ThermoFisher Scientific) for analyzing the peptide mass (MS analysis) and peptide sequence (MS^2^ analysis). HX time-point samples were studied at pD 7.3 and 5°C. All solutions were equilibrated at the desired temperature and HX was initiated by diluting the stock HDL in H_2_O buffer [50 mM (NH_4_)_3_PO_4_, pH 7.3] 10-fold into 100% D_2_O buffer [50 mM (NH_4_)_3_PO_4_, pD 7.3] at 5°C to yield a final apoA-I concentration of 2.5–5 μM. The exchange reaction was quenched at specific time points by adding a precalibrated amount of 99% formic acid on ice to lower the pD to 2.3. A 50 μl aliquot of the quenched apoA-I HX sample was immediately injected into the fragment separation system. Between 0 to 3 min after injection the proteins were digested in the pepsin column and washed in the C18 trap column. The peptides were then roughly separated on an analytical C18 column (flow rate 7 μl/min) with a water-acetonitrile gradient (12–50% acetonitrile) (pH 2.3, 0°C) over 3–15 min. Eluant was injected by electrospray into the spectrometer for mass analysis. Samples containing 100% D to calibrate back-exchange were run and a MS^2^ run on a 100% H sample was performed to calibrate the peptide retention times.

Mass spectra from the HX experiments were analyzed using in-house software ExMS (32). The number of D incorporated at each time point was calculated from the centroid values. This number for each peptide fragment was corrected for back-exchange and used to create a measured HX rate curve (*k*_obs_) for that fragment. The back-exchange was calculated as fraction D retention = (100% D centroid − 100% H centroid) × charge state/number of exchangeable amides in the peptide. The number of deuterons at each HX time point was calculated from the centroid value as, number of D = (centroid of time point − centroid 100% H) × charge state/fraction D retention. The measured HX rate curve was compared with a predicted rate (intrinsic or reference rate, *k*_ref_) curve (33, 34) assuming the fragment to be in a dynamically disordered random coil state so that the amide hydrogens are not protected against exchange with water. The intrinsic rate and observed data were fit to stretched exponentials (16) and a protection factor (Pf) was calculated as a ratio of reference rate/observed rate (Pf = *k*_ref_/*k*_obs_). For more details on data analysis see references (16, 17). Any apoA-I fragment with Pf < 10 is considered to be unprotected (disordered) and any fragment with Pf > 10 is considered to be protected (7, 16). For peptide fragments with an average Pf > 100, the amide hydrogens are involved in hydrogen bonded secondary structure such as α-helix that is relatively stable. Assignment of structure to peptide fragments with Pf in the range 10–100 is ambiguous; such values can arise from either random coil structure that is not completely dynamically disordered or very unstable α-helices that are unfolded for a significant fraction of the time, as shown in other protein systems (35, 36). Pf was used to calculate the free energy of HX (Δ*G*_HX_ = −RT ln (*k*_obs_/*k*_ref_) = RT ln Pf) which corresponds to the free energy of concerted helix unfolding and is therefore a measure of helix stability.

## RESULTS

### Characterization of spherical LpA-I and discoidal HDL particles

**Fig. 1.**
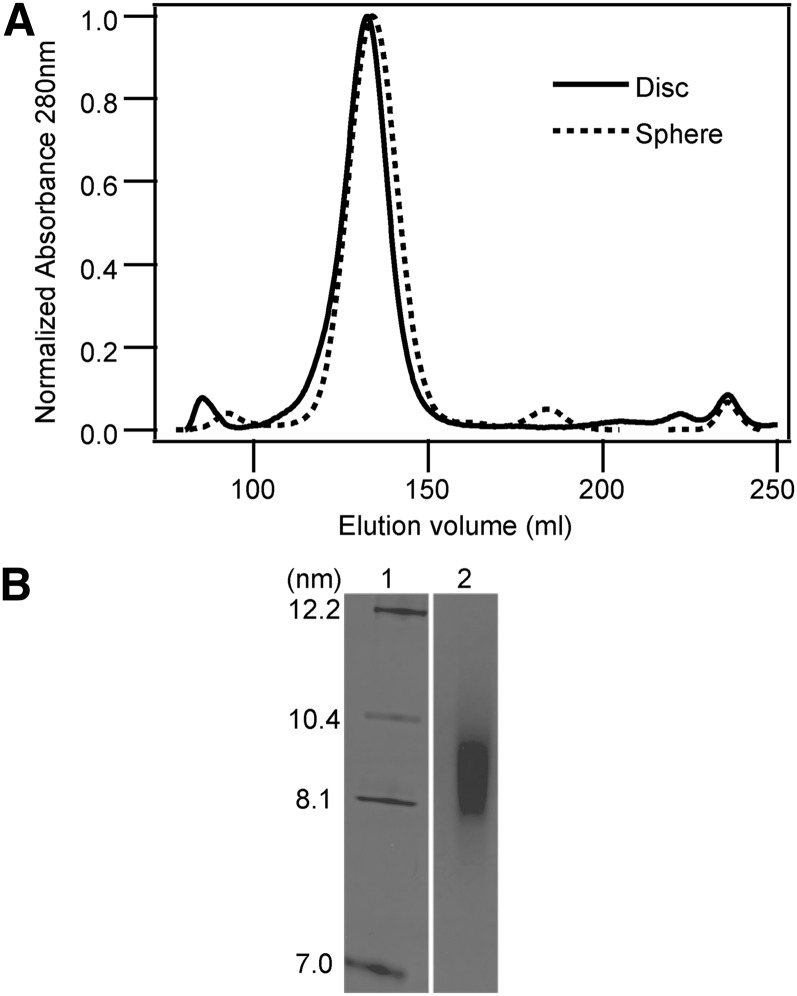
Gel filtration elution profiles of discoidal and spherical HDL particles. A: Reconstituted discoidal HDL particles and plasma spherical HDL (LpA-I) samples isolated by covalent chromatography on thiopropyl Sepharose were purified by gel filtration chromatography using a calibrated Superdex HR200 column. B: Nondenaturing gradient gel electrophoresis of the spherical LpA-I HDL particle preparation.

Homogeneous preparations of approximately 10 nm hydrodynamic diameter discoidal HDL and the spherical LpA-I HDL were isolated by size exclusion chromatography (**Fig. 1**). The central part of the HDL peak was pooled and used for HX experiments. Both types of particles migrated as single bands when analyzed by nondenaturing gradient gel electrophoresis; by this criterion, the hydrodynamic diameter of the spherical particle is 9.4 ± 0.5 nm (Fig. 1B) and the diameter of the discoidal particle is 9.6 ± 0.2 nm [Fig. S1 in (7)]. The ratio of PL to protein in each type of HDL particle is listed in **Table 1**. The composition (% w/w, average of two preparations that differed by <5%) of the spherical particle is as follows: protein 54, PL 30, cholesterol 1, cholesteryl ester 13, and triglyceride 2. The PL composition (% w/w) is phosphatidylcholine 78, sphingomyelin 12, lyso-phosphatidylcholine 4, and acidic PL 5.

**TABLE 1.** Characteristics of spherical LpA-I and discoidal POPC/apoA-I HDL particles

Parameter	Spherical LpA-I	POPC/apoA-I Disc*^a^*
Hydrodynamic diameter (nm)*^b^*	10	10
Phospholipid/apoA-I (w/w)*^c^*	0.56	1.9 ± 0.2
apoA-I mol/particle*^d^*	5	2

a Data from (7).

b Estimated (±0.5 nm) by elution volume from a calibrated gel filtration column (Fig. 1). Average of two LpA-I preparations. The diameters estimated by nondenaturing gradient gel electrophoresis are presented in the text.

c The ratio for two preparations of spherical HDL is an average that agreed to within ±5%.

d Determined by chemical cross-linking, SDS-PAGE (Fig. 2) and MALDI-TOF MS.

The number of apoA-I molecules in each discoidal or spherical HDL particle was estimated by cross-linking with BS^3^ (**Fig. 2**, Table 1). In agreement with previous reports, based on the molecular weight estimate of cross-linked apoA-I, there are two molecules of apoA-I per discoidal particle. The positions of the cross-links in the apoA-I from discoidal HDL yields dimers that migrate at positions corresponding to 56 and 65 kDa during SDS-PAGE (Fig. 2, lane 2). Analysis by MALDI-TOF MS showed that the molecular mass of the cross-linked dimer was 56,965 Da (data not shown). BS^3^ cross-linking of the spherical 10 nm HDL particle yields one major band at 160 kDa corresponding to 4–6 (average 5) molecules of apoA-I per particle (Fig. 2, lane 3); this estimate is in agreement with previously reported values (9). The molecular mass of the peak corresponding to the pentamer of cross-linked apoA-I is 142,490 Da by MALDI-TOF MS (data not shown). Also in agreement with prior characterization of spherical LpA-I particles (9), the estimated areas occupied by the apoA-I and PL components present in the surface of the particle are similar to the surface area deduced from the particle diameter. The discoidal and spherical HDL preparations have α-helix contents of approximately 80 and 76%, respectively, as measured by CD (**Fig. 3**) and are in agreement with prior reports (7, 9).

**Fig. 2.**
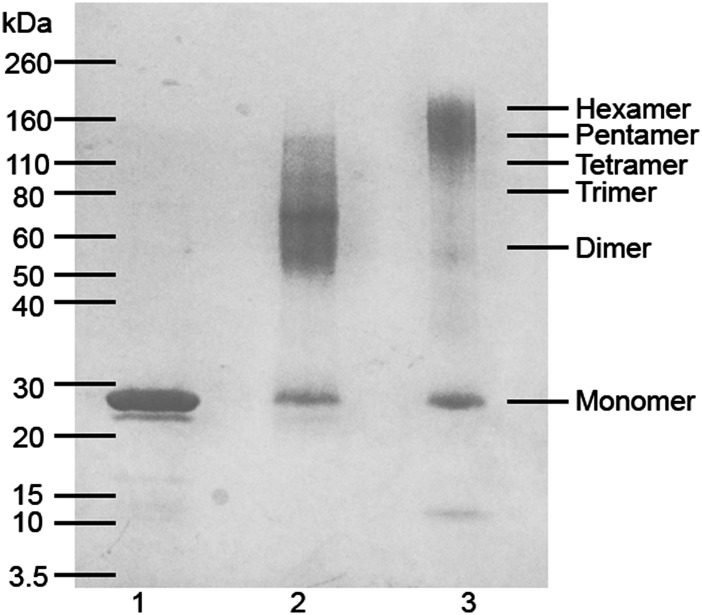
Estimation of the number of apoA-I molecules in 10 nm discoidal and spherical HDL particles. ApoA-I molecules in 10 nm discoidal and spherical HDL particles were cross-linked with 50-fold molar excess of the cross-linker BS^3^. The non-cross-linked lipid-free apoAI and cross-linked HDL samples were analyzed in a 4–20% SDS-PAGE gel. Lane 1, control lipid-free apoA-I without cross-linker; lane 2, cross-linked 10 nm discoidal HDL; and lane 3, cross-linked 10 nm spherical HDL. The migration positions of the molecular mass markers are indicated on the left and the positions of cross-linked apoA-I oligomers are on the right.

**Fig. 3.**
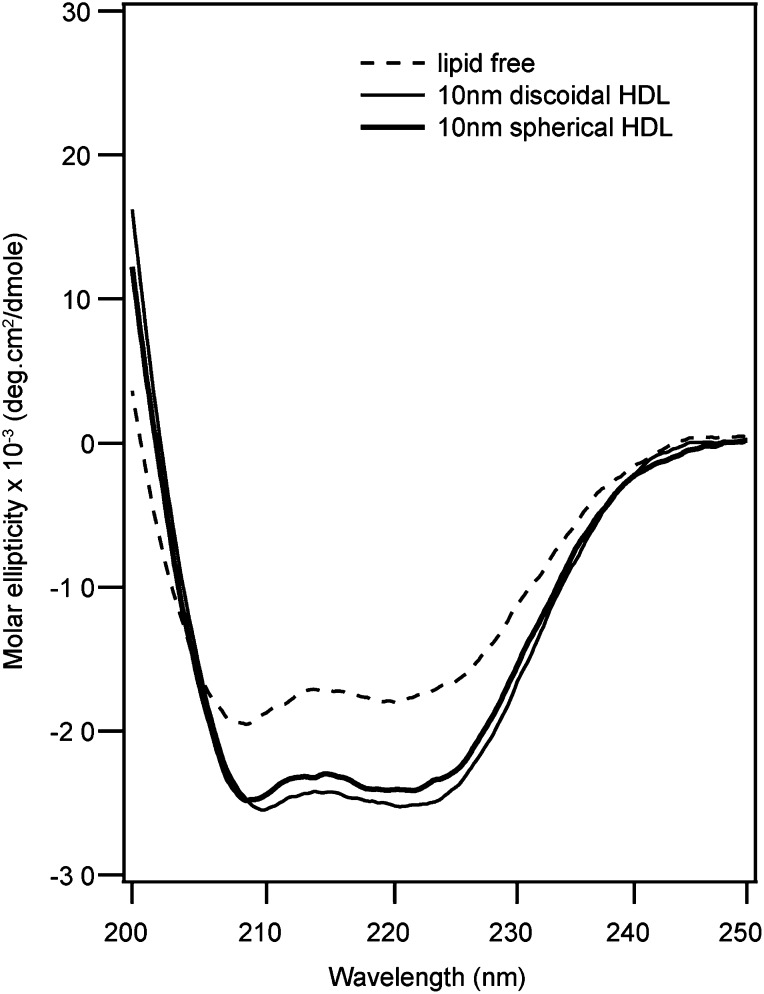
Far-ultraviolet CD spectra of lipid-free apoA-I and lipid-bound apoA-I in 10 nm discoidal and spherical HDL (pH 7.3, 25°C). Analysis of the spectra using Selcon 3 (http://dichroweb.cryst.bbk.ac.uk/html/home.shtml) indicated 52, 80, and 76% α-helix (estimated error ±3%) for lipid-free, 10 nm discoidal, and 10 nm spherical HDL-associated apoA-I, respectively.

### HX-MS of spherical LpA-I and discoidal HDL particles

**Fig. 4.**
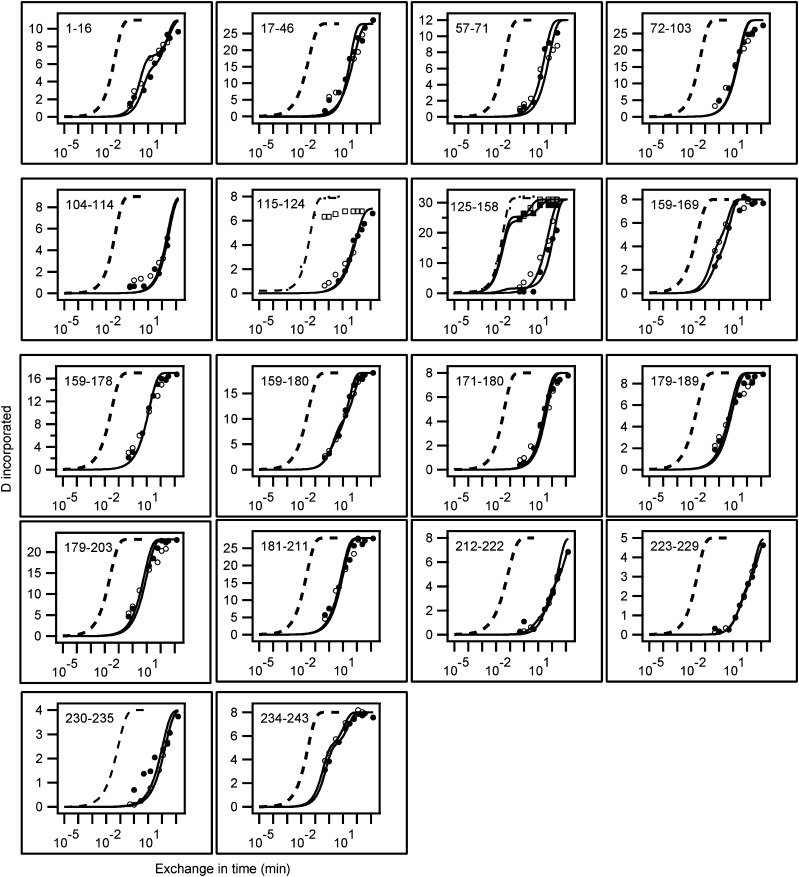
Comparison of HX kinetic profiles of 10 nm discoidal and spherical HDL-bound apoA-I molecules. HX kinetics (pD 7.3, 5°C) for 18 peptides covering the entire sequence of apoA-I molecule (except residues 47–56 for which a fragment in spherical HDL could not be detected) are shown. In each panel (residue numbers are indicated in the top left corner) the observed HX kinetics of an apoA-I fragment from discoidal HDL (•) [data from (7)] and spherical HDL (○) are compared with the intrinsic rate for the peptide. The intrinsic rate is the theoretically computed HX rate of the dynamically disordered random coil with Pf = 1. The time courses are fitted to either mono-exponential or bi-exponential rate equations. The former fit indicates a single cooperatively unfolding segment of secondary structure and the latter fit indicates a peptide that spans a helix terminus (7, 16). Additional time courses (■, discoidal HDL; □, spherical HDL) are shown for peptides between residues 115–124 and 125–158 because the HX-MS envelopes measured for these peptides (Fig. 5) exhibit bimodal behavior.

Previously, we applied HX-MS methodology to define apoA-I helical structure and stability when the protein is in either a lipid-free state (16, 17) or associated with PL in discoidal HDL particles (7). As explained in detail in these publications, the rate of exchange of amide hydrogens with water hydrogens is dependent upon the level of structural protection arising from hydrogen bonding of the amide hydrogens to other groups in the protein. Thus, the HX rates of amide hydrogens involved in α-helix formation in the apoA-I molecule are orders of magnitude slower than the rate for amide hydrogens located in dynamically fluctuating random coil regions of the protein (16). Quantitative measures of these structural effects on HX rate are obtained from the Pf values which are determined from time courses of HX for amides at known positions throughout the protein, as described in the Experimental section. **Figure 4** compares such HX kinetic curves for 18 peptides corresponding to segments covering the length of the apoA-I molecule when the protein is located in 10 nm discoidal and spherical HDL particles. Inspection of these time courses indicates that the rates of HX in most segments of the apoA-I molecule are unaffected by the change in shape of the HDL particle from disc to sphere. This conclusion is supported by the similarities of the Pf values (**Table 2**) for apoA-I segments in the two types of particles. The HX of most peptide fragments is described satisfactorily by a single Pf reflecting the presence of a stable helix that unfolds as a single cooperative unit. In contrast, the terminal peptides 1–16 and 234–243 exhibit bi-exponential HX time courses and have two Pf values indicating that these segments of the apoA-I molecule cross a helix boundary. The numbers of amides located in the two phases of the bi-exponential curve fitting (Table 2) indicate that terminal residues 1–6 and 237–243 are nonhelical and that the remainder of the segments are helical (7). The Pf value gives a measure of helix stability and the free energies of helix stabilization derived in this fashion are in the range 3–5 kcal/mol for HDL particles with both shapes. The fact that most apoA-I peptide fragments listed in Table 2 yield unimodal HX kinetics indicates that all the apoA-I molecules in the spherical HDL particle adopt the same average secondary structure.

**TABLE 2.** HX kinetics of apoA-I peptides in 10 nm spherical HDL particles

Residues	Total Amides	Amides in Phase I	Amides in Phase II	Intrinsic Rate (min^−1^)	Stretch Factor	Pf-I	Pf-II
1–16	11	4	7	21.99	0.83	51	6,718
1–18	13	5	8	20.53	0.84	77	3,606
17–37	19			26.93	0.71	1,984	
17–46	28			27.06	0.74	1,845	
19–46	26			29.26	0.74	1,701	
29–44	14			41.12	0.79	1,593	
29–46	16			36.33	0.78	1,068	
38–46	7			29.84	0.84	958	
57–71	12			23.36	0.84	334	
72–103	29			20.67	0.81	897	
83–103	18			18.12	0.80	1,111	
92–103	9			19.18	0.89	3,367	
93–103	8			18.37	0.88	3,110	
104–114	9			21.25	0.86	6,640	
115–124 fast state	7			21.10	0.73	1	
115–124 slow state	7			21.10	0.73	1,562	
125–158 fast state	31	27	4	30.65	0.79	1	2,513
125–158 slow state	31			30.65	0.79	4,844	
159–169	8			35.85	0.70	110	
159–170	9			28.80	0.68	91	
159–174	13			31.78	0.72	355	
159–176	15			31.80	0.74	440	
159–178	17			31.34	0.76	539	
159–180	19			28.56	0.76	608	
160–170	8			26.63	0.67	106	
165–178	12			28.16	0.78	741	
170–178	7			35.22	0.85	1,497	
170–180	9			28.13	0.82	1,132	
171–180	8			25.38	0.85	911	
179–189	9			37.23	0.74	539	
179–203	23			39.10	0.76	780	
180–189	8			46.34	0.78	564	
181–211	28			38.82	0.78	791	
189–221	29			26.59	0.73	2,456	
190–202	11			55.57	0.84	228	
190–203	12			48.41	0.80	291	
190–205	14			46.57	0.81	277	
190–211	19			38.83	0.80	681	
190–212	20			34.95	0.78	912	
190–213	21			33.30	0.78	812	
190–214	22			30.48	0.76	1,022	
190–219	27			29.74	0.75	2,110	
190–222	29			25.98	0.73	2,635	
191–211	18			41.38	0.81	648	
199–219	18			23.58	0.78	1,754	
203–212	7			21.24	0.87	1,100	
203–214	9			17.91	0.87	1,177	
203–219	14			20.39	0.79	2,163	
203–222	16			17.14	0.77	2,637	
203–224	18			17.31	0.76	2,773	
204–219	13			19.97	0.77	2,234	
204–222	15			16.63	0.76	2,536	
209–224	13			13.77	0.73	1,256	
212–219	6			20.36	0.68	3,847	
212–222	8			14.14	0.68	2,928	
213–222	7			15.97	0.67	4,493	
214–219	4			25.63	0.64	6,250	
214–222	6			14.23	0.64	4,798	
223–229	5			34.10	0.82	4,111	
226–230	3			30.93	0.78	3,934	
226–232	5			42.13	0.80	4,887	
230–235	4			13.54	0.74	851	
233–242	8	5	3	29.30	0.78	7	477
233–243	9	5	4	26.90	0.78	4	178
234–242	7	4	3	36.34	0.81	1	304
234–243	8	5	3	31.80	0.81	10	314
236–243	6	6	0	35.31	0.79	27	

The HX time courses (pD 7.3, 5°C) of apoA-I peptide fragments in 10 nm spherical HDL particles were fitted with either stretched mono-exponential or bi-exponential rate equations and the estimated Pfs are tabulated. The experimental reproducibility of the Pf values is ±20%. If the fit was a mono-exponential equation there is one Pf value and if the best fit was a bi-exponential equation there are two Pf values, one for the fast initial phase (phase I) and one for the second slower phase (phase II). See the Experimental section for more details on curve fitting. The corresponding data for a 10 nm discoidal HDL particle are listed in Table S1 of (7).

**Fig. 5.**
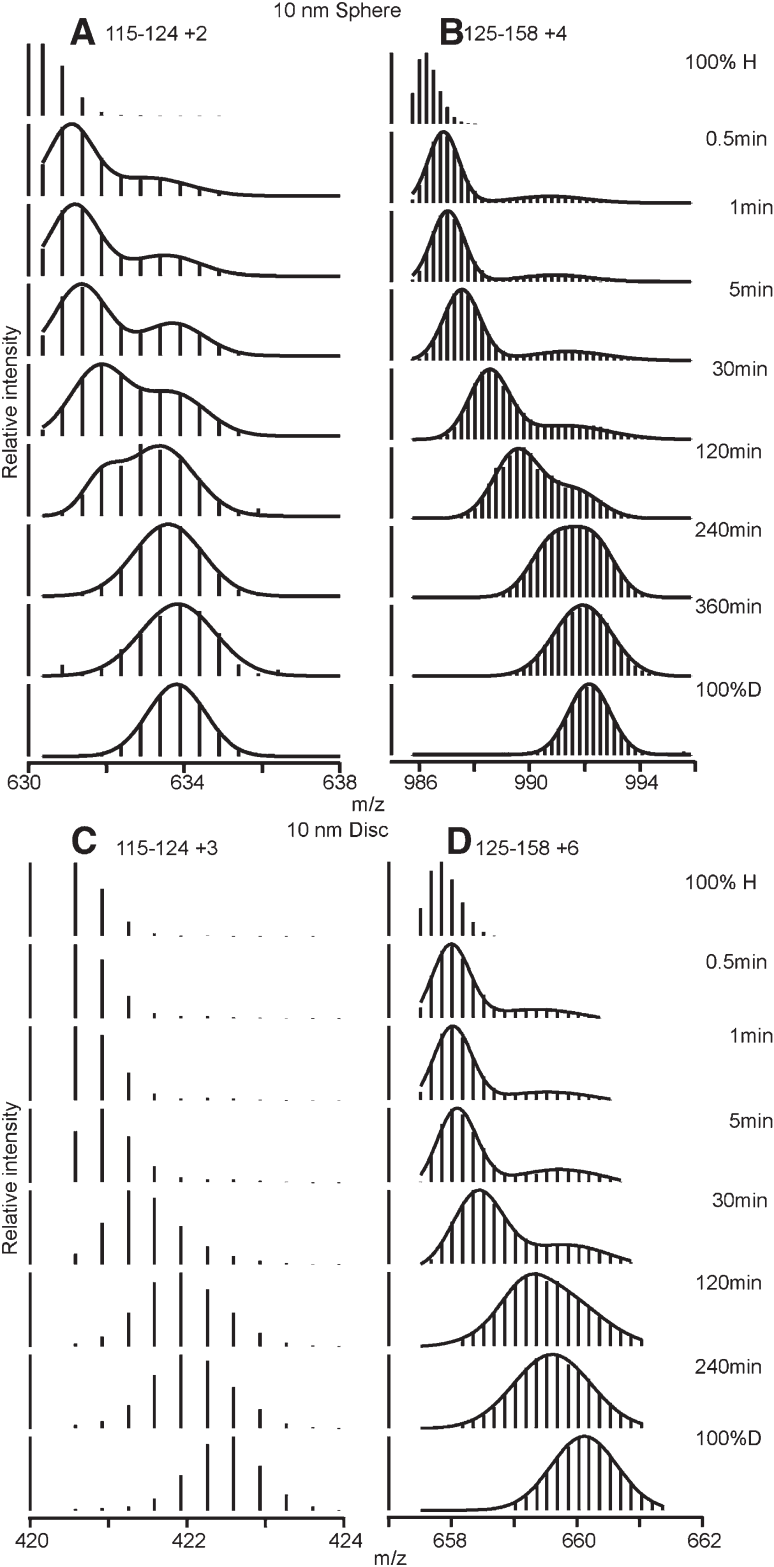
Mass spectra of apoA-I peptides that show bimodal HX kinetics (pD 7.3, 5°C) in 10 nm spherical and discoidal HDL particles. Spherical HDL: (A) peptide 115–124 (charge state +2) and (B) peptide 125–158 (charge state +4). Discoidal HDL: (C) peptide 115–124 (charge state +3) and (D) peptide 125–158 (charge state +6). The mass spectra for peptide fragment 125–158 from discoidal and spherical HDL at HX times between 30 s and 30 min are bimodal indicating that the intact protein exists as two conformations with fast and slow HX rates and therefore different degrees of D incorporation. To estimate the fractions of each population in the HX time-point samples, the mass spectra were fitted to a double Gaussian equation (solid curves in panels A, B, and D) and integrated to obtain peak intensities. For peptide 125–158, at the earliest 30 s time point there are two populations with occupancies of 10–20% (fast state) and 80–90% (slow state), which is an indication of the equilibrium distribution between the two states. The fact that the two populations eventually merge at long HX times but before attaining the 100% D condition indicates that the fast and slow states can inter-convert. Because the two populations are not fully merged to form a single population at 120 min it follows that interchange of this segment of the protein between the two conformations is slow on this timescale (tens of minutes) at 5°C. Rapid inter-conversion between the two states would give rise to a unimodal mass spectrum. The fact that the centroid masses and the relative intensities of the two isotopic distributions both change progressively over the 30 s to 240 min period of D incorporation indicates that, at least in part, the reaction follows EX2 kinetics (i.e., the rate of HX is much slower than the rate of helix refolding). Bimodal isotopic distributions can also occur when the rate of helix refolding is slower than the rate of HX and the reaction follows EX1 kinetics (7, 17). However, if pure EX1 kinetics applied, over the time of HX the *m/z* values of the two isotopic distributions would be expected to remain constant as the relative intensities of the two peaks change. In the case of peptide 115–124, bimodal HX kinetics are seen for the 10 nm spherical HDL particle (A) but unimodal kinetics indicating a single conformation are seen for this segment of the apoA-I molecule in the 10 nm discoidal HDL particle (C).

The peptide corresponding to fragment 125–158 of apoA-I in the 10 nm discoidal HDL particle exhibits bimodal HX kinetics consistent with the occurrence of two populations of intact peptide possessing fast and slow rates of deuterium incorporation (**Fig. 5**). Similar bimodal HX behavior has also been observed for apoA-I located in discoidal complexes formed with dimyristoyl phosphatidylcholine (37). This segment of the apoA-I molecule is distributed in a ratio of up to 4/1 between the slowly and rapidly exchanging populations with exchange to the fully deuterated state not being complete in 240 min. The time courses for these two fast and slow states are shown in Fig. 4 and their Pf values are listed in Table 2. As discussed before (7), this behavior is consistent with the region of residues 125–158 in the apoA-I molecule in a 10 nm discoidal HDL particle occupying coexisting helical and disordered states. As explained in the legend to Fig. 5, interchange between the two states occurs on the timescale of tens of minutes. Similar bimodal HX kinetics are observed for segment 125–158 of apoA-I molecules located in the 10 nm spherical LpA-I HDL particle (Figs. 4, 5 and Table 2). However, in contrast to the situation with the discoidal HDL where fragment 115–124 exhibits unimodal HX kinetics consistent with the existence of only helical structure, this segment of apoA-I in the spherical HDL particle exhibits bimodal HX kinetics (Fig. 5), consistent with formation of coexisting helical and disordered states. It follows that the central segment of the apoA-I molecule that exists in two conformations is elongated to encompass residues 115–158 in the spherical HDL particle. The Pf values (Table 2) for peptides corresponding to this region of the apoA-I molecule are consistent with the fast state being desorbed, disordered loop and the slow state being helix bound to the HDL particle surface (7).

## DISCUSSION

**Fig. 6.**
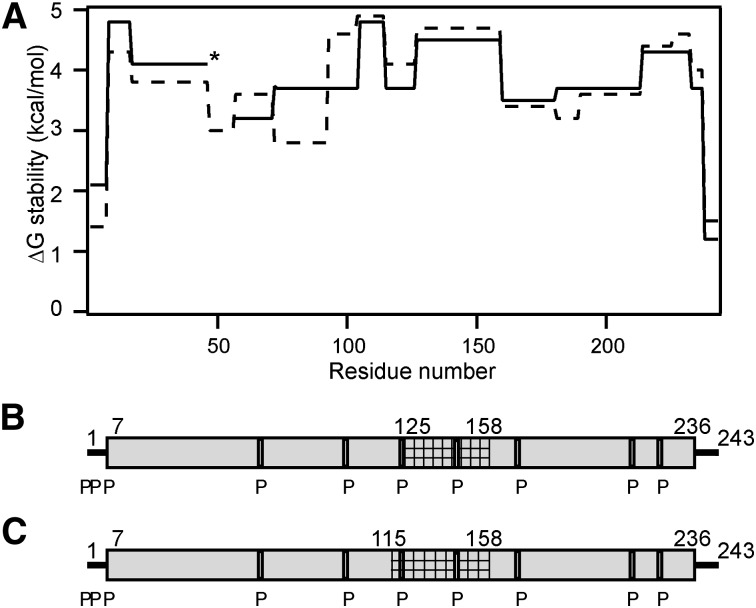
Summary of the HX-derived secondary structure assignments and α-helix stabilities for apoA-I in 10 nm discoidal and spherical HDL. A: Site-resolved stabilities for apoA-I in discoidal (dashed line) and spherical HDL [solid line; the asterisk indicates the gap in coverage for peptide 47–56 (compare Fig. 4)]. The HX kinetic data (pD 7.3, 5°C) for the 10 nm discoidal and spherical HDL particles in Fig. 4 and Table 2 were analyzed to obtain Pf values for each apoA-I peptide fragment. The free energies of stabilization derived from the Pf values are plotted for 17 peptide fragments (represented by a series of connected horizontal lines) that span the length of the apoA-I amino acid sequence. Residues 115–124 (spherical HDL only) and 125–158 [crosshatched in (B) and (C)] exhibit bimodal HX kinetics (see Fig. 5) indicating two populations undergoing HX at different rates; the Δ*G* stability values for the fast state are included in panel (A) (the estimated values are zero and ∼1 kcal/mol for apoA-I in the spherical and discoidal HDL particles, respectively). Panels (B) and (C) compare the HX-derived helix locations in discoidal and spherical HDL particles, respectively. The disordered loop structure formed by the region around residues 125–158 is not included in panels (B) and (C). The gray cylinders represent α-helices and the lines indicate disordered secondary structure. The positions of proline residues (P), whose presence leads to some perturbation of α-helix organization, are marked.

In prior work we determined by HX-MS the helix locations and stabilities in the two apoA-I molecules arranged as a double-belt around the edge of discoidal HDL particles (7). Nearly the entire apoA-I molecule adopts helical structure (except for terminal residues 1–6 and 237–243) when incorporated into a 10 nm disc and these helices have stabilities in the range 3–5 kcal/mol (**Fig. 6A, B**). These free energies of stabilization are relatively low and indicate that the helical segments undergo dynamic unfolding and refolding in seconds or less (7, 16). The comparison of the site-resolved stabilities (Fig. 6A) and locations (Fig. 6C) for apoA-I in 10 nm discoidal and spherical HDL particles indicates that helix structure is similar in both types of particles.

The apoA-I segment spanning residues 125–158 in a 10 nm disc exhibits bimodal HX kinetics (Fig. 5) indicating coexisting helical and disordered loop conformations that interchange on a timescale of tens of minutes. When the disc diameter is decreased to ∼8 nm so that the area at the disc edge available to each apoA-I molecule is decreased by 20%, the segment involved in this helix-loop conformation is extended to include residues 115–158 (7). The same increase in length of this segment occurs in 10 nm spherical LpA-I particles (Fig. 6C), as indicated by the bimodal HX kinetics of peptide 115–124 (Fig. 5). This increased participation in loop formation (with amino acids protruding into the aqueous phase) is consistent with an increased apoA-I packing density on the surface of the spherical HDL particle. Such an increase in packing density does indeed occur. Thus, the 10 nm sphere and disc contain averages of five and two apoA-I molecules, respectively (Table 1), and the surface area in the former particle is only approximately twice the available area around the edge of the disc (assuming that the thickness of the segment of PL bilayer forming the disc is 5 nm). Strikingly, the resultant ∼20% decrease in surface area available per apoA-I molecule on the spherical LpA-I particle induces residues 115–124 to adopt a coexisting helix and loop structure, which is precisely the same conformational change seen when the hydrodynamic diameter of discoidal particles is reduced from 10 to 8 nm (7). These findings are consistent with the concept that the coexisting helix-loop configuration is sensitive to apoA-I surface packing density, thereby allowing the protein to adjust to different size discoidal and spherical HDL particles. Such variations in loop formation are probably the reason for the altered lysine microenvironments (12) and protease sensitivity (14) found for apoA-I molecules in the two differently shaped HDL particles.

Overall, the fact that apoA-I helix characteristics on discoidal and spherical HDL particles are similar is consistent with the finding that the double-belt organization is largely maintained on both types of particles. The trefoil structure (8, 9) [and related structures that accommodate this concept (10)] adopted by the apoA-I molecule forms a dynamic scaffold (helices unfolding and refolding in seconds) that controls the size of the spherical HDL particle. The ability of the central region encompassing residues 115–158 to adopt an inter-converting helix-loop conformation presumably provides a means for the apoA-I molecules to adjust to variations in packing density that result from alterations in particle size. This finding is consistent with prior antibody binding evidence that the conformation of the apoA-I segment spanning residues 121–165 is particularly sensitive to changes in spherical HDL particle size (38). Twisting of the apoA-I molecules to various degrees may also occur on HDL particles of different sizes (9). In addition to such changes in conformation, reductions in spherical HDL particle size can lead to dissociation of apoA-I molecules (39, 40).
